# Hypogonadal Rescue in Post-orgasmic Illness Syndrome: A Case Report

**DOI:** 10.7759/cureus.92552

**Published:** 2025-09-17

**Authors:** Nora A Alrebdi, Fahad K Alanazi, Maan A Alsania, Mohammed A Almosa

**Affiliations:** 1 Urology, King Saud Medical City, Riyadh, SAU

**Keywords:** andrology, andro urology, hormonal therapy, male sexual health, pois, post-orgasmic illness syndrome

## Abstract

Post-orgasmic Illness Syndrome (POIS) is a debilitating and poorly understood condition characterized by multi-system symptoms following ejaculation. We report a hormonally responsive POIS phenotype treated with clomiphene citrate and niacinamide. A 36-year-old Middle Eastern man (BMI 24.5) fulfilling all six Waldinger diagnostic criteria for POIS underwent baseline endocrine profiling and symptom assessment, including serum testosterone, estradiol, luteinizing hormone (LH), follicle-stimulating hormone (FSH), and thyroid-stimulating hormone (TSH). He was treated with clomiphene citrate 50 mg daily and niacinamide 500 mg daily for three months, with monthly safety monitoring. Pretreatment testosterone was 10.7 nmol/L (normal 8.6-29.0), estradiol 114 pmol/L (41-159), and total symptom score 62/90. After three months, testosterone normalized to 41.9 nmol/L and estradiol rose to 229 pmol/L, with a 26% reduction in total symptom score and a decrease in episode duration from 12 to nine days. No adverse events or laboratory abnormalities occurred. Targeted endocrine modulation with clomiphene and niacinamide produced a clinically meaningful reduction in symptom burden and episode duration in this POIS case, supporting routine hormonal assessment and personalized therapy. Prospective, controlled trials employing validated outcome measures are warranted to establish efficacy and safety.

## Introduction

Post-orgasmic Illness Syndrome (POIS) is a rare and frequently underrecognized clinical condition first described by Waldinger and Schweitzer in 2002. It is characterized by debilitating physical, cognitive, and psychological symptoms, including flu-like symptoms, fatigue, muscular pain, nasal congestion, cognitive impairments (such as poor concentration and memory difficulties), and mood disturbances (anxiety and irritability), emerging within minutes to hours after ejaculation and lasting several days up to a week [1,2].

The exact pathophysiology of POIS remains unclear. Current hypotheses include autoimmune or allergic reactions to seminal fluid components, neuroendocrine imbalances, and autonomic dysregulation [2-4]. Its clinical symptoms closely overlap with more common conditions such as chronic fatigue syndrome, depression, generalized anxiety disorder, and allergic disorders, leading to frequent misdiagnosis and delayed interventions [3,4].

Treatment of POIS remains empirical and challenging due to unclear pathogenesis and limited clinical data. Approaches described in existing literature include antihistamines, selective serotonin reuptake inhibitors (SSRIs), hormonal therapies, immunotherapy (such as hyposensitization protocols), and behavioral interventions with variable outcomes [2,3].

Due to limited clinical awareness, POIS continues to pose diagnostic and therapeutic challenges, significantly affecting patients' quality of life. Detailed reporting of clinical cases is essential to enhance recognition, refine diagnostic criteria, and identify effective management strategies. Here, we present a clinical case of a young male diagnosed with POIS, focusing on the diagnostic process, symptom progression, and therapeutic outcomes, aiming to contribute to the clinical understanding and management of this debilitating condition.

## Case presentation

A 36-year-old Middle Eastern man (BMI 24.5) first presented on June 5, 2024, with a 10-year history of POIS, beginning abruptly and severely approximately 10 years ago, with progressively worsening episodes. Immediately following ejaculation, the patient consistently experiences severe lower limb pain, particularly localized posteriorly behind the knees, accompanied by significant weakness radiating toward his back. Subsequently, cognitive disturbances occur, including impaired concentration, noticeable deficits in both short- and long-term memory, and episodes of disoriented speech. These symptoms persist for about ten days before spontaneously resolving without residual effects (Table 1) [2].

**Table 1:** Waldinger diagnostic criteria fulfilled by the patient

Waldinger criterion	Evidence in this case
1. Symptoms occur after ejaculation	Yes
Onset of symptoms	Within 30 minutes
2. Somatic symptom present	Severe lower limb myalgia
3. Cognitive symptom present	Concentration deficit
Cognitive symptom present	Memory deficit
4. Symptom duration	Seven 10 days
5. Occurrence rate	100% of ejaculations (including nocturnal emissions)

Notably, he denies fever or flu-like symptoms typically associated with POIS. Due to symptom severity and the profound negative impact on his marital relationship, resulting in divorce, and his professional life, the patient abstained from all sexual activity, including masturbation, for the past five years. Nevertheless, nocturnal emissions continue to trigger symptom recurrence.

His past medical history includes appendectomy and nasal polypectomy with no psychiatric history. Physical examination revealed a generally healthy appearance with normal secondary sexual characteristics. Genital examination demonstrated bilateral Grade II varicocele, with both testes of normal size and consistency. The right spermatic cord appeared contracted and was notably tender on palpation.

Baseline laboratory investigations on 05-06-2024 revealed the following: testosterone 10.72 nmol/L, prolactin 7.47 μg/L, estradiol 114.00 pmol/L, follicle-stimulating hormone (FSH) 2.60 IU/L, luteinizing hormone (LH) 2.17 IU/L, and thyroid-stimulating hormone (TSH) 1.27 mIU/L (Table 2).

**Table 2:** Patient baseline and follow-up laboratory findings with normal values included

Laboratory parameter	Baseline values	After three month follow-up values	Normal range
Testosterone	10.72	41.93	8.64-29.0 nmol/L (adult male)
Estradiol	114.00	229.00	41-159 pmol/L (adult male)
Prolactin	7.47	6.30	4.04-15.2 μg/L
Follicle-stimulating hormone (FSH)	2.60	5.09	1.5-12.4 IU/L
Luteinizing hormone (LH)	2.17	5.96	1.7-8.6 IU/L

Therapeutically, the patient was administered clomiphene citrate 50 mg orally once daily and niacinamide 500 mg daily, with antihistamines, over a three-month duration. Clomiphene citrate, an estrogen receptor modulator, was intended to increase endogenous testosterone production, while niacinamide was employed for its potential anti-inflammatory and vasodilatory effects. Three-month follow-up laboratory evaluations showed significant hormonal improvement: testosterone rose to 41.93 nmol/L, estradiol increased to 229.00 pmol/L, prolactin decreased slightly to 6.30 μg/L, LH rose to 5.96 IU/L, FSH increased to 5.09 IU/L, and TSH slightly decreased to 0.91 mIU/L (Table 2).

Symptom improvement was evaluated solely by subjective patient reporting, indicating a modest reduction in symptom duration from 12 to nine days per episode, and the patient estimated a 5% decrease in overall symptom severity after a three-month course; no adverse side effects were reported. 

“Even a partial reduction in my ‘brain fog’ lets me function at work. I hope future research brings a cure.”

Despite significant anxiety and occupational impairment, the patient declined psychological counseling. Written informed consent for publication of this case report was obtained. Continued counseling and endocrine treatment are recommended, with further hormonal assessments planned during follow-up visits.

## Discussion

This case highlights the diagnostic and therapeutic challenges associated with POIS, a rare and underdiagnosed condition first described by Waldinger and Schweitzer in 2002 [1]. While typically characterized by flu-like symptoms, fatigue, and cognitive dysfunction, our patient presented with a classical yet atypical constellation, lacking systemic symptoms like fever or myalgia.

The recurrence of symptoms following nocturnal emissions, despite complete cessation of voluntary sexual activity, reinforces the hypothesis of a neurohormonal or immunogenic mechanism rather than purely psychogenic etiology. Several pathophysiological mechanisms have been proposed, including type I or IV hypersensitivity reactions to seminal antigens [1,2], autoimmune responses [3], and autonomic dysregulation [4]. The patient's baseline hypogonadism and estradiol elevation raise the possibility that endocrine dysregulation may play a contributory role, as suggested by previous observations linking low testosterone to symptom flares in POIS [5].

Empirical therapy remains the cornerstone of POIS management due to a lack of evidence-based guidelines. Our use of clomiphene citrate, an estrogen receptor antagonist that promotes endogenous testosterone production, resulted in normalization of androgen profiles, aligning with similar case reports indicating symptom relief following hormonal therapy [6]. Niacinamide was added for its vasoprotective and anti-inflammatory properties, and may have contributed to subjective improvement, although its use in POIS is novel and requires further validation.

Although symptom relief was modest, a reduction in episode duration from 12 to nine days and ~5% decrease in perceived severity, these improvements are clinically relevant in the context of POIS’s debilitating nature. This aligns with previous studies where even partial responses significantly improved quality of life [2,5]. The lack of validated outcome tools, however, continues to hinder standardized assessment of treatment efficacy, underscoring the need for prospective studies employing structured symptom scales.

Despite significant psychosocial consequences, including occupational impairment requiring job resignation, marital dissolution resulting in divorce, and depression stemming from diminished quality of life. The patient declined psychiatric intervention. Multidisciplinary management involving endocrinology, allergy/immunology, and mental health services has been advocated in the literature for comprehensive care [4].

This case demonstrates the evolving clinical landscape of POIS by documenting a unique response to endocrine-targeted therapy. It highlights the potential utility of clomiphene and niacinamide in hormonally mediated presentations and underscores the importance of individualized approaches in the absence of standardized guidelines. Continued documentation of such cases is essential to guide future research and inform evidence-based management strategies.

## Conclusions

This case highlights the clinical complexity and heterogeneity of POIS, which can manifest without classical systemic flu-like symptoms and thereby challenge traditional diagnostic patterns. The observed hormonal abnormalities, coupled with symptomatic improvement under clomiphene citrate and niacinamide, suggest that endocrine modulation may represent a therapeutic option in selected POIS phenotypes.

These findings emphasize the importance of individualized, hormone-informed management strategies in patients for whom conventional approaches are limited or ineffective. By documenting a hormonally responsive phenotype, this report contributes a novel therapeutic perspective and underscores the need for systematic, collaborative research to validate treatment strategies and improve quality of life in affected individuals.
